# Supplementary material for: Decreased susceptibility to viscosin in Streptococcus pneumoniae
Source: Microbiol Spectr. 2024 Jul 3;12(8):e00624-24. doi: 10.1128/spectrum.00624-24 (PMC11302323; doi:10.1128/spectrum.00624-24)
Supplement: Supplemental table and figures — Table S1; Fig. S1-S5. [file spectrum.00624-24-s0001.pdf]

Supplemental material

Decreased susceptibility to viscosin in *Streptococcus pneumoniae*.

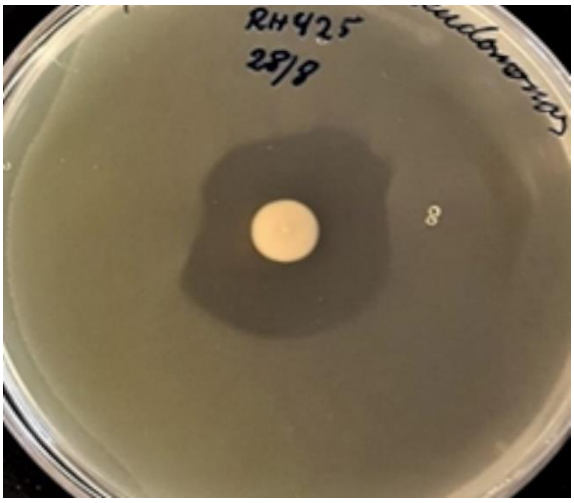

Fig. S1. Soft-agar overlay assay showing inhibition zone of *S. pneumoniae* surrounding a macrocolony of *Pseudomonas* sp. Three µl of an overnight culture of *Pseudomonas* sp. was spotted on TH-agar and incubated for 48 hours at room temperature. Five mL TH soft-agar (45 °C) containing 100 µl of *S. pneumoniae* RH425 at OD_{550} = 0.3 were poured on top of the macrocolony. The plate was incubated anaerobically at 37 °C overnight.

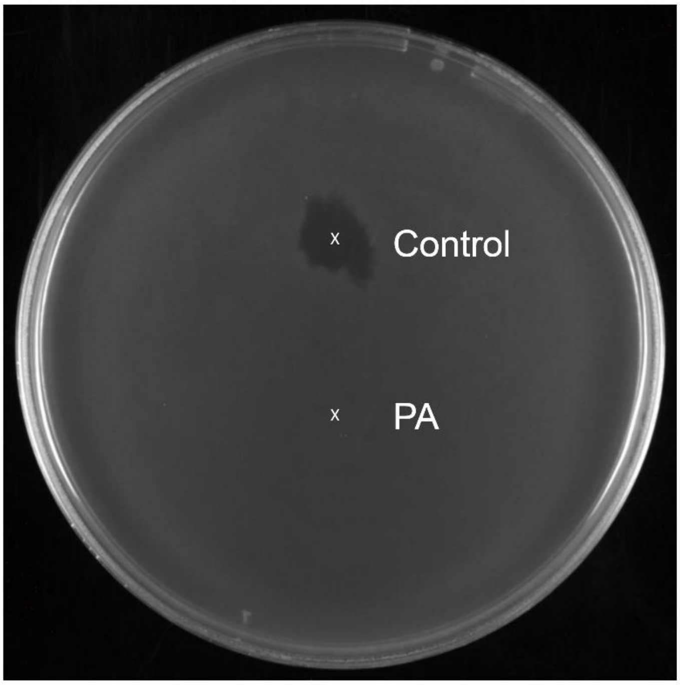

Fig. S2. Soft-agar overlay assay showing that polymyxin acylase inactivated the antimicrobial activity of the compound purified from *Pseudomonas* sp. culture supernatants, in line with being a lipopeptide. Five µg of the antimicrobial compound were treated with 2.5 µg polymyxin acylase (PA) at 37 °C for 18 hours and then spotted onto a soft-agar layer containing *S. pneumoniae* RH425. Compound without polymyxin acylase was treated similarly as control. The plate was incubated at 37 °C overnight. Growth inhibition is seen as a clearing zone on the spotted area (indicated with x).

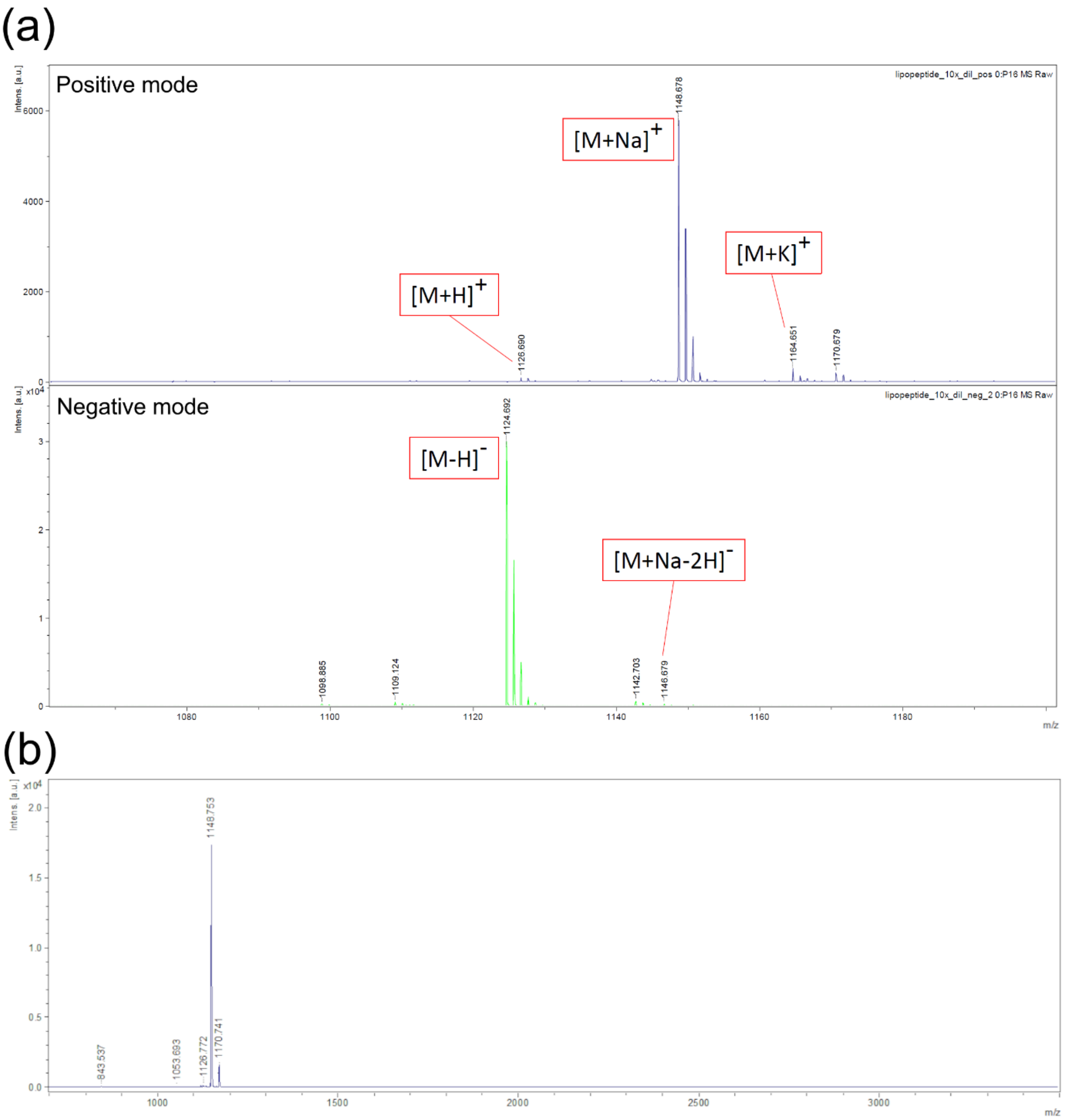

Fig. S3. MALDI-TOF MS analysis of the antimicrobial material eluting after 17 minutes in the HPLC chromatogram of Fig. 1b. (a) The ion signals m/z 1126.7, 1148.7 and 1164.7 correlated with the weight of the protonated molecular ion and sodium and potassium adducts of viscosin ($[M+H]^+$ = 1126.4). Analysing the compound in the negative mode detected an ion signal at m/z 1124.7 corresponding with the mass of the $[M-H]^-$ -ion. (b) Ion signals obtained in the detection range m/z 700-3500.

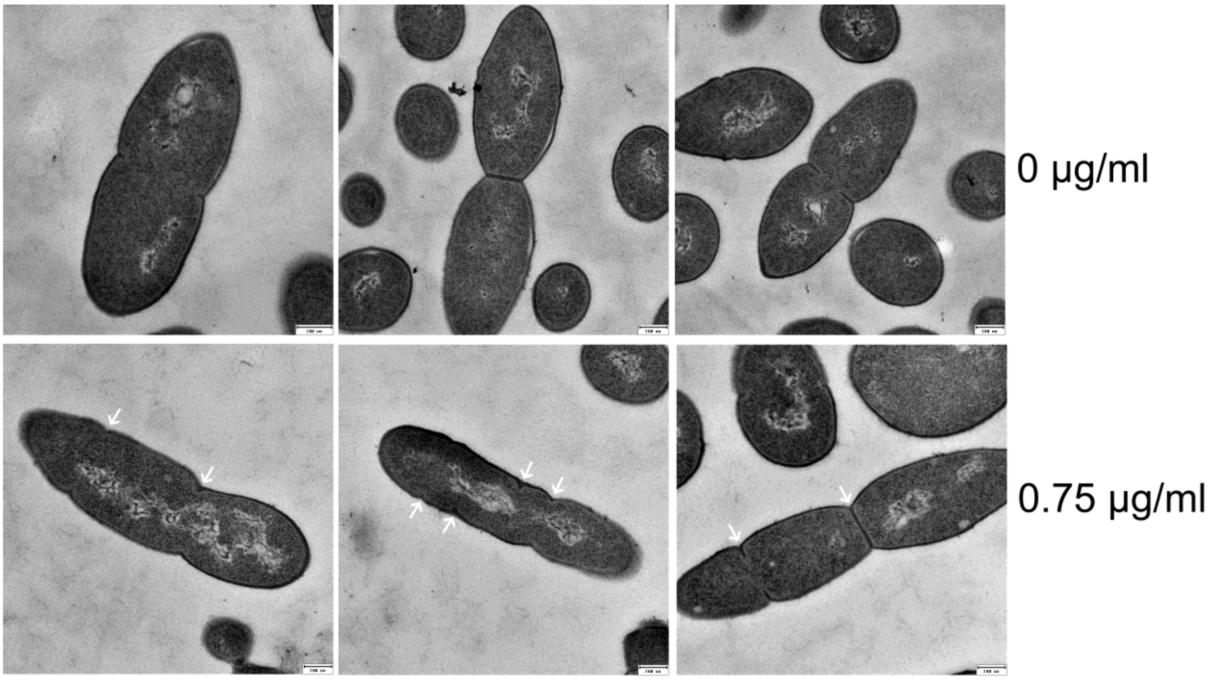

Fig. S4. Transmission electron microscopy images of *S. pneumoniae* RH14 (Δ*lytA*) grown in Todd Hewitt broth containing a final concentration of 0 and 0.75 µg/ml daptomycin (corresponds to 1.5 - 3x MIC_{90} reported for *S. pneumoniae* (1, 2)) for four hours. Ca^{2+} ($CaCl_2$) was added to a final concentration of 1.25 mM to both non-treated and treated cultures. Arrows indicate incomplete septa and cross-wall splitting. Scale bars, 200 nm.

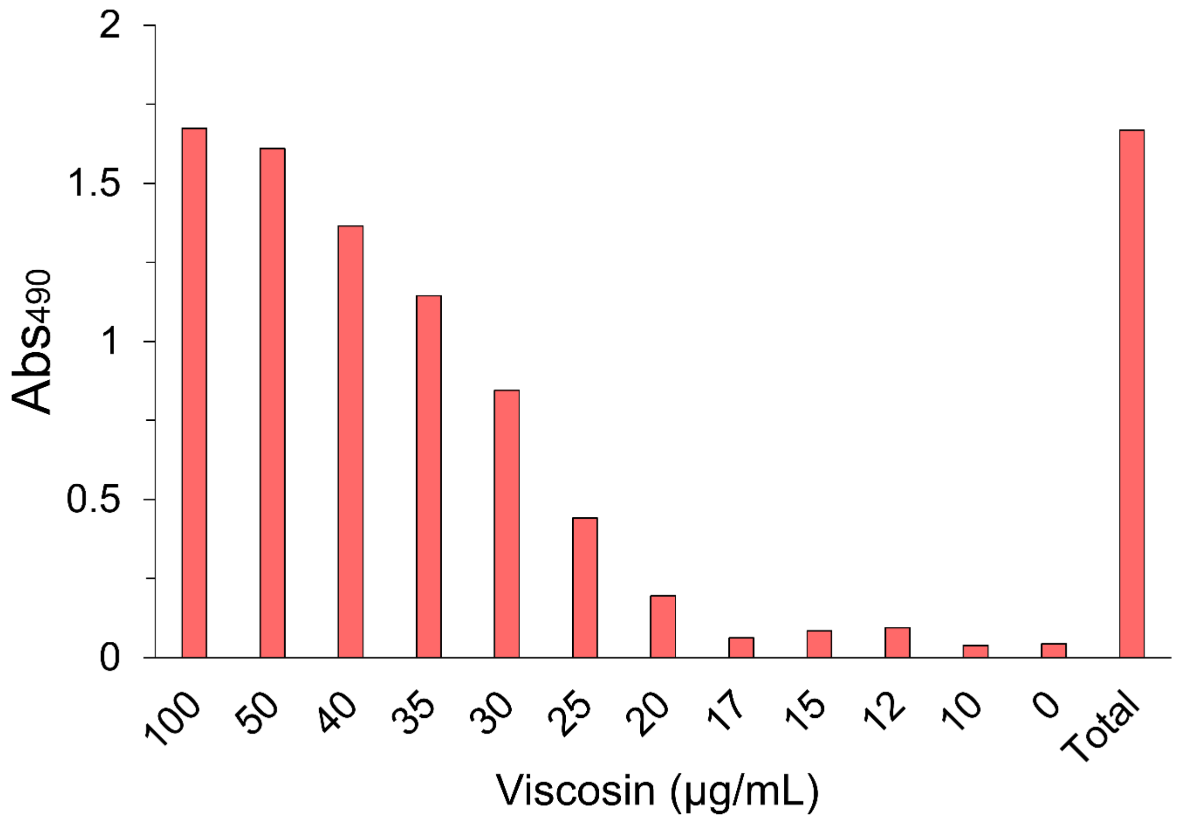

Fig. S5. Hemolysis test of viscosin. The final concentrations of viscosin added to erythrocytes diluted 1:9 in PBS are indicated on the X-axis. Total hemolysis was obtained by adding a final concentration of 1% Triton-X100.

Table S1. Primers used in this work.

Oligo Name	Sequence 5’-3’		Reference
KHB50	CCGATGCAGAAATGGTTGAG		(3)
KHB51	GATAATCGTACATCTGAAGCTC		(3)
KHB53	TCATTGTAAGCGCCCAATAAC		(3)
KHB78	ATGAGATCCGCCAAAAACATA		(4)
aw214	GAGCTTCAGATGTACGATTATCAG AGTTATCCACAGTTTGTGTAAA		This work
aw215	CTTTATGTTTTTGGCGGATCTCATA TTTGCCTCCATATGTTTGAATTAC		This work

References.

1. Piper KE, Steckelberg JM, Patel R. In vitro activity of daptomycin against clinical isolates of Gram-positive bacteria. J Infect Chemother. 2005;11(4):207-9.
2. Pankuch GA, Jacobs MR, Appelbaum PC. Bactericidal activity of daptomycin against *Streptococcus pneumoniae* compared with eight other antimicrobials. J Antimicrob Chemother. 2003;51(2):443-6.
3. Berg KH, Biørnstad TJ, Straume D, Håvarstein LS. Peptide-regulated gene depletion system developed for use in *Streptococcus pneumoniae*. J Bacteriol. 2011;193(19):5207-15.
4. Stamsås GA, Straume D, Salehian Z, Håvarstein LS. Evidence that pneumococcal WalK is regulated by StkP through protein-protein interaction. Microbiology (Reading). 2017;163(3):383-99.
